# Siderophore Scaffold as Carrier for Antifungal Peptides in Therapy of *Aspergillus fumigatus* Infections

**DOI:** 10.3390/jof6040367

**Published:** 2020-12-15

**Authors:** Joachim Pfister, Roland Bata, Isabella Hubmann, Anton Amadeus Hörmann, Fabio Gsaller, Hubertus Haas, Clemens Decristoforo

**Affiliations:** 1 Department of Nuclear Medicine, Medical University Innsbruck, 6020 Innsbruck, Austria; joachim.pfister@i-med.ac.at (J.P.); roland.bata@student.uibk.ac.at (R.B.); isabella_hubmann@gmx.at (I.H.); anton.hoermann@i-med.ac.at (A.A.H.); 2 Institute of Molecular Biology, Medical University Innsbruck, 6020 Innsbruck, Austria; fabio.gsaller@i-med.ac.at (F.G.); hubertus.haas@i-med.ac.at (H.H.)

**Keywords:** *Aspergillus fumigatus*, infection, invasive pulmonary aspergillosis, siderophores, TAFC, antifungal peptide, PAF26, theranostics, gallium-68, PET/CT

## Abstract

Antifungal resistance of human fungal pathogens represents an increasing challenge in modern medicine. Short antimicrobial peptides (AMP) display a promising class of antifungals with a different mode of action, but lack target specificity and metabolic stability. In this study the hexapeptide PAF26 (Ac-dArg-dLys-dLys-dTrp-dPhe-dTrp-NH2) and the three amino acid long peptide NLF (H2N-Asn-Leu-dPhe-COOH) were coupled to diacetylfusarinine C (DAFC), a derivative of the siderophore triacetylfusarinine C (TAFC) of *Aspergillus fumigatus*, to achieve targeted delivery for treatment of invasive aspergillosis. Conjugated compounds in various modifications were labelled with radioactive gallium-68 to perform in vitro and in vivo characterizations. LogD, serum stability, uptake- growth promotion- and minimal inhibitory concentration assays were performed, as well as in vivo stability tests and biodistribution in BALB/c mice. Uptake and growth assays revealed specific internalization of the siderophore conjugates by *A. fumigatus*. They showed a high stability in human serum and also in the blood of BALB/c mice but metabolites in urine, probably due to degradation in the kidneys. Only PAF26 showed growth inhibition at 8 µg/ml which was lost after conjugation to DAFC. Despite their lacking antifungal activity conjugates based on a siderophore scaffold have a potential to provide the basis for a new class of antifungals, which allow the combination of imaging by using PET/CT with targeted treatment, thereby opening a theranostic approach for personalized therapy.

## 1. Introduction

The airborne fungus *Aspergillus fumigatus* (*A. fumigatus*) is responsible for local and systemic infectious diseases, especially in immunocompromised patients. A major threat is invasive pulmonary aspergillosis (IPA), a systemic opportunistic infection of the lung with a mortality rate of up to 90%. The reasons for this high lethality rate origin from insufficient diagnostic methods, but even if IPA is diagnosed early, overall mortality of 50% occurs [1]. Therefore, it is of high interest to develop new diagnostic as well as therapeutic options.

A big challenge for *A. fumigatus* during the infection process represents the hostile environment in the host. One of the most important limiting factors of fungal development is iron supply. [2] *A. fumigatus* uses a highly sophisticated siderophore system to overcome this problem and gather iron during infection [3]. In this setting, *A. fumigatus* secretes small organic molecules, so-called siderophores, into its surrounding tissue to trans-chelate iron with high affinity. *A. fumigatus* uses two types of siderophores to gather iron from the environment: triacetylfusarinine C (TAFC) and its precursor fusarinine C (FsC) [4]. After chelating iron, TAFC is reabsorbed by a specific siderophore transporter called MirB [5], while the transporter for [Fe]FsC has not been identified yet. Modifying TAFC using FsC as scaffold allows the creation of molecules that act as specific and target-oriented carriers to the fungal cell [6,7,8]. For this purpose, the diacetylated form of TAFC, diacetylfusarinine C (DAFC) was used as starting material. Its free amine function allows chemical modification, for example, the coupling of carboxylic acids by a common peptide coupling strategy.

**Figure 1:**
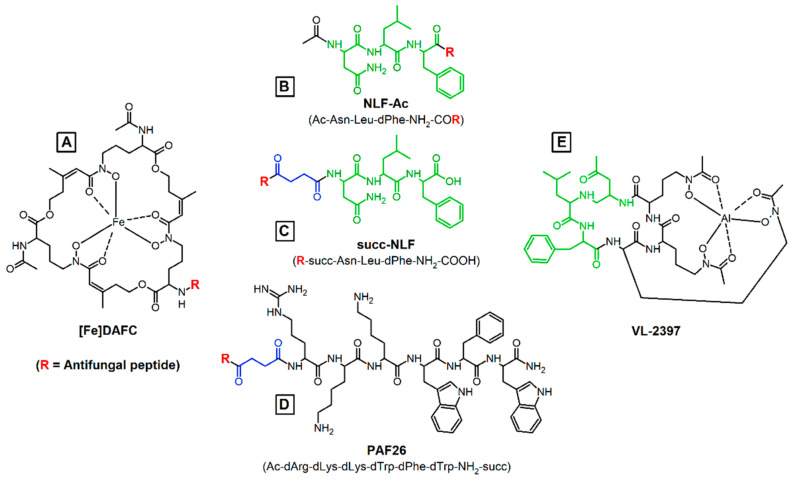
Chemical structures of the siderophore (**A**) [Fe]DAFC with its free amino group for coupling and conjugation of antifungal peptides. NLF peptide with two different modifications: (**B**) NLF-Ac directly coupled via carboxylic group and acetylated amino end; (**C**) succ-NLF with a free carboxylic acid and at the N-terminus succinyl group for coupling. (**D**) PAF26 in both variants, D-PAF26 (original) and L-PAF26 (for control), were also coupled via succinyl-linker to the amine of [Fe]DAFC. (**E**) The ferrichrome analog VL-2397 with the antifungal “backbone” in green. Red:Coupling site; Blue: succinylic spacer.

Natural or synthetically produced antimicrobial peptides represent a promising new class of compounds for therapy of infectious diseases [9]. Different modes of action potentially allow overcoming the increasing problem of resistance in microorganisms against conventional therapeutic strategies [10]. Antifungal peptides have been intensively investigated to develop drugs with novel mechanisms of action [11]. López-García et. al. discovered the hexapeptide PAF26 (Ac-rkkwfw-NH_2_; Figure 1) containing only D-amino acids, with reported antifungal activity against various different microorganisms [12] and especially for the human pathogen *A. fumigatus* [13,14]. The mode of action of these small peptides is not fully understood until now but they show a discrete penetration into the hyphal cell, accumulate in the vacuoles and are then released into cytosol followed by cell death [15]. Muñoz et. al. showed that modification of the N-terminus of PAF26 with fluorescent dyes is possible without losing activity and cell penetration properties [16], which forms the basis for further modification to improve antifungal properties. In this study, we also synthesized PAF26 with L-amino acids as a control to get a better understanding of antifungal activity and pharmacokinetic properties.

A second peptide, which was investigated in this study, is the 3 amino acid peptide “NLF” (H2N-Asn-Leu-dPhe-COOH). It represents the most likely antifungal portion of the recently identified antifungal siderophore VL-2397 [17], which exhibits high activity against *A. fumigatus* [18]. Compared to the non-toxic siderophore ferrichrome (FC), three glycine residues are replaced by Asn-Leu-dPhe in VL-2397. Therefore, we intended to investigate the potential antifungal activity of this tripeptide alone as well as coupled to a carrier molecule.

Conjugation of such antifungal peptides to the FsC scaffold potentially also allows radioactive labelling for diagnostic purposes by means of Positron Emission Tomography (PET) using Gallium-68 [19]. This enables to plan and personalize potential treatment. Such a “theranostic” concept has recently attracted great interest in the field of oncology [20,21,22] but has not been applied in the field of fungal infections. The specificity of TAFC for *A. fumigatus* enables the deliverance of conjugated molecules target-specific to the fungus and also decreases the applied drug amount to the patient [7]. Additionally, the circulation time of the applied conjugate in the bloodstream could be reduced due to this specific uptake system and therefore reduce the effect of metabolic instability, which is an especially important aspect of using peptides in drug therapy [23].

The aim of this study was to synthesize peptides, couple them to the FsC scaffold using DAFC and evaluate them regarding their in vitro and in vivo properties including characterization of their antifungal activity. Finally, we aimed to provide a proof of concept of using siderophores as carriers for antifungal peptides enabling theranostics in fungal infections. 

## 2. Materials and Methods

### 2.1. Synthesis of Antifungal Conjugates

The synthesis of precursor substances and antifungal peptides are presented in detail in the Supplementary Material section. Briefly, peptides were produced by solid-phase peptide synthesis (SPPS) using rink amide resin and 2-Chlortrityl resin [24]. For conjugation of the individual peptides a O-(7-Azabenzotriazol-1-yl)-N,N,N’,N’-tetramethyluronium-hexafluorphosophat (HATU) coupling strategy was selected to bind the peptides to the free amine group of [Fe]DAFC, or using the carboxylic group of [Fe]DAFC succ [8]. Conjugates were purified using preparative RP-HPLC and the identity was confirmed by MALDI-TOF MS. 

For radiolabelling, iron was removed from the complex by incubation with a 1000-fold excess of ethylenediaminetetraacetic acid (EDTA) at pH 4 and subsequent purification by preparative RP-HPLC.

### 2.2. Radiolabelling

Fractionated elution of ^68^Ge/^68^Ga-generator (IGG100. Eckert and Ziegler Isotope Products, Berlin, Germany; nominal activity of 1850 MBq) with 0.1 M hydrochloric acid (HCL, Rotem Industries, Arva, Israel) was used to obtain ^68^GaCl3 (~250 MBq) in 1 mL eluate. For labelling, 10 μg (5–8 nmol) of DAFC-conjugate were mixed with 200 μL gallium eluate (~15–30 MBq) and the pH was adjusted to 4.5 by adding 20 μL of sodium acetate solution (1.14 M) per 100 μL eluate. The mixture was left to react for 10 min at RT and finally analyzed by radio-TLC (Scan-RAM™, LabLogistic, Sheffield, UK) and radio-RP-HPLC.

### 2.3. In Vitro Characterization

#### 2.3.1. Distribution Coefficient

Aliquots of 50 µL of ^68^Ga-labelled conjugate (~5 µM) were added to 450 µL of phosphate-buffered saline pH 7.4 (PBS) and 500 µL of Octanol into a 1.5 mL Eppendorf tube. The mixture was vigorously shaken for 20 min at 1400 rpm in a standard vortex (MS 3, IKA, Staufen, Germany) and hereafter, 200 µL of each phase removed and measured in a gamma counter (2480 automatic gamma counter Wizard2 3’’, Perkin Elmer, Waltham, MA, USA) LogD was calculated as the ratio of octanol/water phase using Excel. (*n* = 3, six technical replicates)

#### 2.3.2. Protein Binding

To determine protein binding an aliquot of 50 µL ^68^Ga-labelled conjugate (~5 µM) was added to 450 µL of fresh human serum or 450 µL of PBS (as control) and incubated at 37 °C for 30, 60 and 120 min. At each time point 25 µL were removed and analyzed by size exclusion chromatography using MicroSpin G-50 columns (Sephadex G-50, GE Healthcare, Vienna, Austria) according to the manufacturer’s protocol. Column and eluate were measured in the gamma counter and the percentage between protein-bound (eluate) and free conjugate (column) was calculated. (*n* = 3)

#### 2.3.3. Serum Stability

Similar to protein binding preparation, ^68^Ga-labelled conjugate was added to 450 µL of fresh human serum or 450 µL of PBS (as control) and incubated at 37 °C for 60, 120 and 240 min. At each time point 70 µL were removed and mixed with 70 µL of acetonitrile to precipitate all proteins. The supernatant was then analyzed by radio-RP-HPLC to investigate free gallium and metabolites.

#### 2.3.4. Uptake and Competition Assay

Assays were performed as previously described [8,25]. Briefly, uptake and competition assays were performed with *A. fumigatus* in iron-depleted and iron-replete *Aspergillus* minimal media (AMM) [26], using a 96-well MultiScreen Filter Plates HTS (1 μm glass fiber filter, Merck Millipore, Darmstadt, Germany). Compounds were incubated for 45 min at 37 °C and filters measured in a gamma counter. Competition assays were performed in the same way except for pre-incubation with the iron-containing antifungal conjugates for 15 min, resulting in a competition at the MirB transporter to show specific binding to MirB.

#### 2.3.5. Growth Promotion Assay

To assess the capability of *A. fumigatus* to utilize the modified siderophores, a mutant strain (*ΔsidA/ΔftrA*) that lacks the genes *sidA* and *ftrA* was used. These mutations impair both siderophore-mediated iron acquisition and reductive iron assimilation, in other words, high-affinity iron acquisition [27]. If the activity of externally supplied siderophore was still intact, similar growth to [Fe]TAFC should be observed, however, in the case of growth reduction, it cannot be distinguished from antifungal activity. Spores (10^4^) were point inoculated on solid 0.5 mL of AMM [26] in 24-well plates containing increasing concentrations of iron labelled siderophore ranging from 0.1–50 μM. Plates were incubated for 72 h at 37 °C in a humidity chamber and visually assessed [6]. 

#### 2.3.6. Minimal Inhibitory Concentration (MIC) Assay

Susceptibility tests were performed according to CLSI guidelines with slight modifications. In brief, 96-well plates (Greiner Bio-One GmbH) were prepared with 100 μL of 2 × AMM (*Aspergillus* minimal medium [26]) or 0.2 × Potato Dextrose Broth (PDB) containing 3 × 10^4^ spores of *A. fumigatus* (ATCC 46645) per well. An amount of 100μL antifungal, dissolved in water, was added to get a final concentration of 1 × AMM/0.1 × PDB medium and antifungal concentration ranging from 0.031–16 μg/mL. The lowest concentration without visible growth was used to determine the MIC value after incubation for 24 and 48 h at 37 °C.

### 2.4. In Vivo Characterization

All animal experiments were conducted in compliance with the Austrian animal protection laws and with the approval of the Austrian Ministry of Science (BMWFW-66.011/0161- WF/V/3b/2016).

#### In Vivo Stability and Ex Vivo Biodistribution

Stability test and biodistribution were conducted in 4–6 weeks-old female BALB/c mice (in-house breed, ZVTA Innsbruck). ^68^Ga-labelled conjugates, dissolved in PBS (Ph. Eur. pH 7.4) and pH adjusted to pH 6.5 with sodium acetate, were injected via lateral tail vein using approximately 0.4 nmol of compound and a volume of 150 µL.

To assess the stability, mice were injected with radiolabelled conjugate (~12 MBq) and euthanized after 10 min by cervical dislocation. Blood/urine were collected and immediately analyzed by radio RP-HPLC. Proteins in blood samples were precipitated by adding 0.1% TFA/ACN (1:1 *v/v*) and the supernatant was used for further analysis with radio RP-HPLC. The percentage of intact conjugate was calculated by integration of the radio-chromatograms.

For biodistribution, the same procedure was applied except for different time points of 45 and 90 min. Hereafter the animals were euthanized by cervical dislocation and the organs (blood, spleen, pancreas, stomach, liver, kidneys, heart, lung, muscle, femur) were removed and weighed. The activity of the samples was measured in a gamma counter and the results were calculated as a percentage of injected dose per gram tissue (%ID/g).

## 3. Results

### 3.1. Synthesis and Radiolabelling

The synthesized peptides and conjugates are displayed in Figure 1. Peptide synthesis and conjugation to the siderophore were achieved with high chemical purity (80–90% monitored by analytical RP-HPLC). The corresponding mass analysis confirmed the calculated values. Radiolabelling with the modified siderophore was achieved within 10 min at room temperature, with quantitative radiochemical yields (>95%).

### 3.2. In Vitro Characterization

#### 3.2.1. LogD, Protein Binding and Serum Stability

All compounds showed a high hydrophilicity with logD values of around −3.0 except for acetylated peptide DAFC-NLF-Ac with −0.80. Protein binding was very low for the short peptides, whereas elongation of the amino acid chain also increases protein binding values to more than 20%. Stability tests in human serum revealed very high stability for DAFC-NLF-Ac and succ-NLF with no formation of degradation products. [^68^Ga]Ga-DAFC-L-PAF26 showed considerable decomposition of the conjugate over time whereas no degradation was detected for [^68^Ga]Ga-DAFC-D-PAF26 (Table 1).

#### 3.2.2. Uptake and Competition Assay

**Figure 2:**
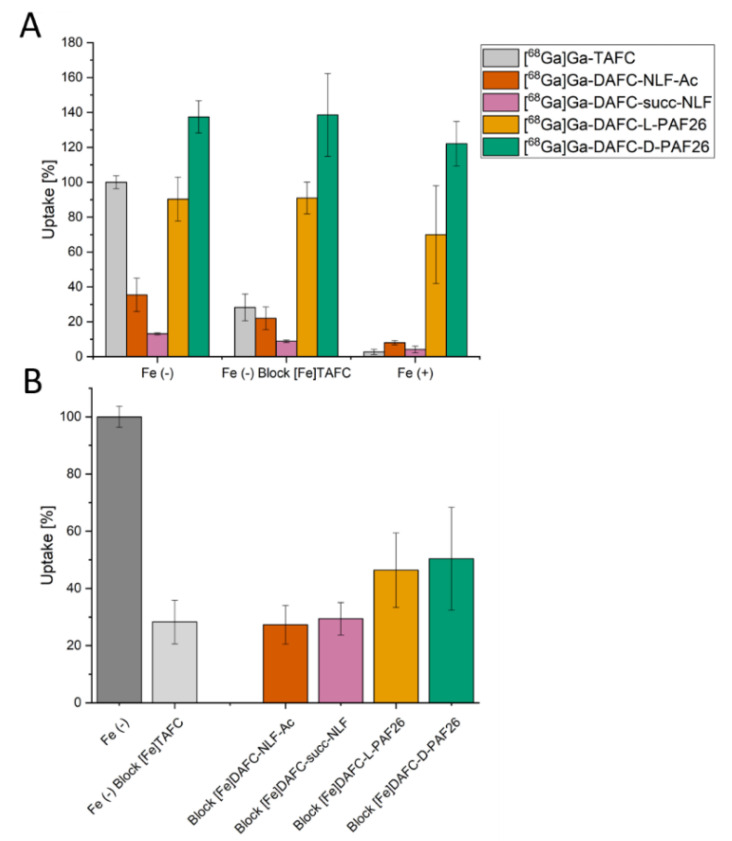
(**A**) Uptake of radiolabelled conjugates normalized to uptake of [^68^Ga]Ga-TAFC of each experiment, respectively. Grey bars represent control with [^68^Ga]Ga-TAFC. Uptake can be blocked with [Fe]TAFC due to competition for uptake by the MirB transporter. Iron-repleted conditions [Fe (+)] lead to a decrease in MirB biosynthesis and active uptake into the hyphae. (**B**) Competition assay of [^68^Ga]Ga-TAFC blocked with iron-containing antifungal conjugates in iron-depleted fungal culture. For all compounds a reduction of [^68^Ga]Ga-TAFC uptake could be observed, indicating a specific interaction with the MirB transporter.

Uptake and competition studies in *A. fumigatus* are summarized in Figure 2. All values shown are normalized to the uptake of [^68^Ga]Ga-TAFC of each experiment, respectively. Uptake should decrease when blocked with an excess of [Fe]TAFC or in iron-sufficient media, causing transcriptional repression of siderophore uptake [28]. This behavior could be observed for [^68^Ga]Ga-DAFC-NLF-Ac and succ-NLF. In contrast [^68^Ga]Ga-DAFC-L-PAF26 and D-PAF26 showed almost the same uptake under blocking and iron-sufficient conditions. [^68^Ga]Ga-D-PAF26 showed even higher uptake values than [^68^Ga]Ga-TAFC, possibly related to discrete penetration of the hyphae.

Nevertheless, in the competition assay of iron labelled antifungal conjugates with [^68^Ga]Ga-TAFC, a decrease in TAFC uptake was observed, confirming interaction with the MirB transporter, whereby NLF conjugates showed more efficient blocking than PAF26.

#### 3.2.3. Growth Promotion Assay

**Figure 3:**
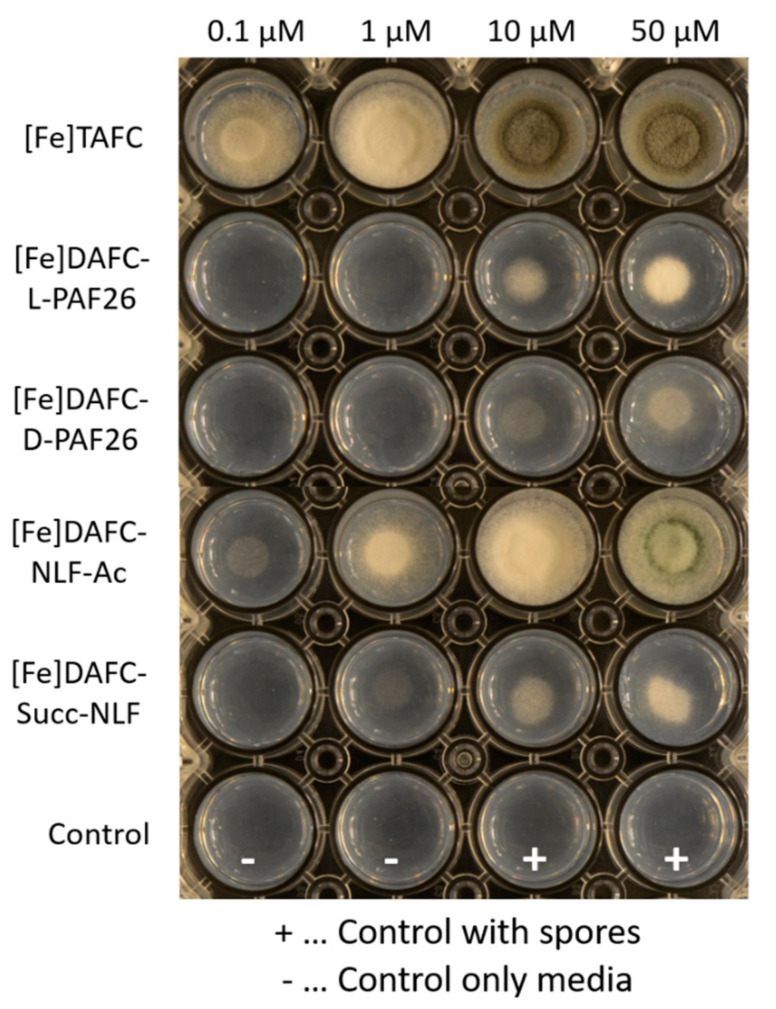
Growth promotion of *A. fumigatus* mutant strain *ΔsidA/ΔftrA* after 72 h incubation at 37 °C on iron-depleted *Aspergillus* minimal media (AMM) with different concentrations of iron labelled conjugates. Whitish hyphal growth can be distinguished from greenish sporulating growth due to the green pigmentation of conidia. The bottom row shows control without siderophores.

Utilization experiments showed for [Fe]TAFC (control) growth induction at 0.1 µM and sporulation at 10 µM. (Figure 3). [Fe]DAFC-NLF-Ac resulted in a similar growth rate and also sporulation at 50 µM. In contrast [Fe]DAFC-succ-NLF could be utilized by *A. fumigatus* only at 10–50 µM and with a very low growth promotion, possibly related to its negative charge of the carboxylic acid. Both compounds [Fe]DAFC-L-PAF26 and -D-PAF26 resulted in a low hyphal growth without sporulation, whereby L-PAF26 could be utilized slightly better. Taken together, these data indicate that iron from these antifungal conjugates can be utilized by *A. fumigatus*, but the length and charge of the peptide are playing a decisive role. 

#### 3.2.4. Minimal Inhibitory Concentration (MIC)

To evaluate the antifungal potential of the new conjugates, MIC assays were performed according to the modified CLSI guidelines. Compounds were tested in two different media which varied from the standard RPMI media. *Aspergillus* minimal medium (AMM) without iron addition has a very low iron content that forces the fungus to upregulate its siderophore system, comparable to a clinical infection, where fungi face low iron availability [2]. Under these conditions, none of the compounds displayed antifungal activity at the tested concentrations (Table 2).

In 0.1 × potato dextrose buffer (PDB), which has been used in the context of antifungal peptides [29], only H_2_N-D-PAF26-NH_2_ peptide showed antifungal activity with a MIC of 8 µg/mL (Table 3).

### 3.3. In Vivo Experiments

#### In Vivo Stability and Biodistribution

10 min after injection of the compound into female BALB/c mice the in vivo stability of siderophore conjugates was determined, in analogy to protocols previously reported [30]. Degradation processes in the blood by enzymes and also metabolism in the kidneys were derived from the radio-HPLC analysis of blood and urine (Table 4). [^68^Ga]Ga-DAFC-succ-NLF showed high stability in blood and only slight decomposition of 10% after excretion through the urinary tract. [^68^Ga]Ga-DAFC-NLF-Ac, the uncharged and more lipophilic compound, revealed stability in the blood of 86% and only 64% in the urine. [^68^Ga]Ga-DAFC-D-PAF26 showed metabolites in the urine with only 57% intact compound but high stability in the blood.

Biodistribution after 45 min and 90 min of the conjugates are shown in Table 5. Differences between [^68^Ga]Ga-DAFC-NLF-Ac and succ-NLF were primarily in the accumulation into intestine and kidneys, respectively. [^68^Ga]Ga-DAFC-NLF-Ac had high kidney retention, which could also explain the higher metabolite rate in the urine, and higher intestinal excretion, probably related to its higher lipophilicity. [^68^Ga]Ga-DAFC-succ-NLF showed very low retention in all organs except for the kidneys, the main excretion route for this hydrophilic compound. [^68^Ga]Ga-DAFC-D-PAF26 showed very high retention of the peptide in the kidneys, correlating with a higher metabolic rate in urine, but also higher levels in the liver. For a better understanding also biodistribution of [^68^Ga]Ga-TAFC was added for comparison, where very low levels of radioactivity in all organs were found and only low kidney retention was observed [31], indicating that attachment of a peptide sequence increases in particular kidney reuptake and retention. 

## 4. Discussion

Fungal infections represent a rising challenge for physicians in all countries of the world [32]. High lethality rates [1], as well as increasing resistance to common antifungals, demand new therapeutic options including the use of peptides with antifungal properties. The antimicrobial peptide PAF26 contains only the D-amino acids, which are important for their antifungal activity. In this study, L-PAF26 was also synthesized as a control. The short peptide NLF was conjugated to DAFC in two different modifications: Via N-terminal acetylation and direct coupling via the carboxylic acid (NLF-Ac) or N-terminally coupled via a succinyl linker to DAFC, resulting in a free carboxylic acid at the end (succ-NLF). The main difference between these compounds represent the charge of the whole molecule, which is neutral for [^68^Ga]Ga-DAFC-NLF-Ac and single negatively charged for [^68^Ga]Ga-DAFC-succ-NLF.

LogD values of these compounds revealed overall hydrophilic molecules, except for [^68^Ga]Ga-DAFC-NLF-Ac, which can be explained by its uncharged character, whereas all other conjugates were charged. In addition, a low protein binding was observed, favoring more rapid renal excretion. Incubation in human serum revealed overall stability for all compounds except for [^68^Ga]Ga-DAFC-L-PAF26 (60 min 38 %; 240 min 14%). [^68^Ga]Ga-DAFC-D-PAF26 showed almost no degradation at all, even after 240 min, which can be expected from a peptide consisting of unusual amino acids not contained in mammalian peptides and proteins. This high stability was confirmed in vivo in the blood of BALB/c mouse, with >99% of [^68^Ga]Ga-DAFC-D-PAF26 remaining intact. In contrast, only 57% of the intact compound was found in the urine sample. This indicates that the main excretion route of the compound and also metabolization occurs in the kidneys of the mouse. This phenomenon has already been observed in earlier studies with radioactively labelled peptides, often showing high retention in the kidneys, due to reabsorption in the tubular system as well as metabolic processes [33]. [^68^Ga]Ga-DAFC-NLF-Ac showed a slight decomposition in the blood of BALB/c mice and only 64% of the conjugate excreted intact in the urine. [^68^Ga]Ga-DAFC-succ-NLF revealed only slight metabolism in the urine samples and no instability in blood at all. This phenomenon could be explained by amino acid order, since the terminal amino acid in DAFC-succ-NLF is D-phenylalanine, which makes the peptide less prone to enzymatic degradation by exopeptidases. These results are underlined by the biodistribution data. A very high kidney uptake for [^68^Ga]Ga-DAFC-D-PAF26 (150 %) indicates an efficient accumulative process in the kidneys, which was also the case for [^68^Ga]Ga-DAFC-NLF-Ac (36%), but at a much lower level. The length as well as the charge of the peptide seems to have a decisive impact on the retention, which remained high even after 90 min and was correlated with a high percentage of metabolized compound in the urine. In contrast, the negatively charged and highly hydrophilic short peptide [^68^Ga]Ga-DAFC-succ-NLF showed only low levels of kidney retention and also considerably lower ratios of metabolites in the urine. For comparison, biodistribution of the unmodified siderophore [^68^Ga]Ga-TAFC showed practically no kidney retention with a very rapid excretion through the urinary tract with no metabolites detected [34].

Uptake assays into *A. fumigatus* hyphae revealed very high radioactivity levels for both compounds [^68^Ga]Ga-DAFC-D-PAF26 and -L-PAF26 with only low reduction when blocked with [Fe]TAFC or in iron sufficient media. This indicates unspecific binding to the cell wall of the hyphae as well as discrete direct uptake of D-PAF26, reflected by the higher uptake of D-PAF26 (137%) vs. L-PAF26 (90%). [^68^Ga]Ga-DAFC-NLF-Ac and –succ-NLF showed considerably lower values of 35% (NLF-Ac) and 13% (succ-NLF), which were significantly reduced by blocking. This difference is probably caused by the negative charge of succ-NLF, a phenomenon that has been observed with similar conjugates as well [6]. Competition assays showed a reduction of [^68^Ga]Ga-TAFC uptake of all compounds, indicating a specific interaction with the MirB transporter. These results were confirmed in growth assays, only high concentrations of both [Fe]DAFC-D/L-PAF26 compounds were able to promote the growth of the *A. fumigatus* mutant strain *ΔsidA/ΔftrA* indicating poorly conserved siderophore activity. In contrast, [Fe]DAFC-NLF compounds showed a better growth promotion and NLF-Ac reached almost the value of [Fe]TAFC; the difference to succ-NLF clearly reflects the uptake results. The generally lower growth promotion of peptide conjugates could also be indicative for some antifungal activity, however, it cannot be distinguished from the growth promotion effects of iron in this assay.

MIC assays were performed according to the CLSI standards, except for the media. We used standard *Aspergillus* minimal medium (AMM) because of its controlled iron content. The siderophore transporter MirB, which is assumed to be essential for a specific action of our siderophore conjugates, is only expressed under iron-depleted conditions [5], which is also the case during infection in the human host [35]. In the literature, an IC50 value of 8 µM after 24 h and 37 °C in 0.1 × Vogel’s medium for H_2_N-D-PAF26-NH_2_ has been reported for *A. fumigatus* [14]. In the current study, no inhibition could be observed in AMM. Only in 0.1 × PDB medium, a clear inhibition at 8 µg/mL could be observed. D-PAF26 contains cationic structures which are similar to other antifungal peptides like PAF B. Notably, these peptides show a different inhibitory effect depending on the medium used [29]. When coupled to [Fe]DAFC, inhibitory effects could neither be observed for [Fe]DAFC-D-PAF26 nor for -L-PAF26 in AMM and 0.1 × PDB. Dietl et. al. showed a MIC value of 1 µg/mL for the antifungal siderophore VL-2397 in RPMI medium after 48 h [18]. Unfortunately, the postulated antifungal moiety of VL-2397, the tripeptide NLF, lacked antifungal potential. Similarly, both derivatives NLF-Ac and succ-NLF lacked antifungal activity up to 16 µg/mL when conjugated to [Fe]DAFC.

Taken together, different peptides with antifungal potential could be coupled to the DAFC molecule and were recognized by the MirB transporter. However, no antifungal activity was observed after coupling to the siderophore. Uptake assays demonstrated that small peptides with up to 3 amino acids can be efficiently taken up dependent on the charge of the molecule. By increasing the length of the amino acid chain, especially when the overall charge is increased, unspecific binding to the hyphae was more pronounced and growth promotion was reduced indicating impaired utilization by *A. fumigatus*. Our studies underline that it is very important also to consider pharmacokinetic behavior (in vivo metabolism, biodistribution and elimination) of antimicrobial peptides as therapeutics, especially in view of potentially high kidney retention of these often highly charged peptides. A previous study investigating the uptake of fluorescence DAFC conjugates in an *A. fumigatus* lung infection model clearly demonstrated in vivo accumulation in the infected area [6]. Therefore, it is very likely that siderophore peptide conjugates would also accumulate in *A. fumigatus* under these physiological conditions. However, with regard to animal welfare and the lack of antifungal activity of the compounds, these experiments have not been performed. Considering the immense arsenal of antimicrobial peptides, selecting more suitable candidates or introducing cleavable linkers to the targeting moiety are strategies to enhance antifungal effects. Besides peptide structures, with potential limitations in size and charge impairing recognition by MirB, conjugation of small molecules (e.g., common antifungals in medicine and agriculture) to DAFC seems an attractive approach to produce more potent antifungal conjugates (improved MIC) but also potentially better biodistribution and uptake behavior. Moreover, we could show that due to the specificity of TAFC-analogs for the MirB transporter [7], selective targeting of *A. fumigatus* is possible. This confirmed our rationale to achieve target-specific accumulation in the fungus by siderophore conjugation and by labelling of the siderophore with, e.g., radioactive gallium-68 localization of the infection by PET/CT can be achieved, thereby combining diagnostic and therapeutic properties in a theranostic approach.

## 5. Conclusions

Overall, this study demonstrates that the concept of coupling antifungal peptides to DAFC is feasible and results in conjugates with retained uptake and recognition by *A. fumigatus*. Radiolabelling was possible under mild conditions, to use these compounds for in vitro and in vivo tests as well as potential diagnostic purposes by PET/CT. Biodistribution studies revealed kidney retention for these peptide-based conjugates with high variability dependent on peptide length and charge, which should be considered in the selection for diagnostic applications. Antifungal activity was lacking with the selected peptide sequences, but overall a proof of concept for the feasibility of antifungal peptide DAFC conjugates was provided. Alternative small peptides or the introduction of cleavable linkers could lead to a new class of antifungals. Additionally, small antifungal molecules can be a promising candidate to overcome the rising problem of antifungal resistance and combined with radiolabeling lead to new theranostic compounds.

## Figures and Tables

**Table 1:** Distribution coefficient, protein binding and serum stability of siderophore compounds radiolabelled with gallium-68. Protein binding is displayed as percentage eluting from size exclusion column (Microspin G-50) after serum incubation, values of serum stability reflect the percentage of intact radiolabelled conjugate.

		[^68^Ga]Ga-DAFC-NLF-Ac	[^68^Ga]Ga-DAFC-succ-NLF	[^68^Ga]Ga-DAFC-L-PAF26	[^68^Ga]Ga-DAFC-D-PAF26
Distribution coefficient *n* = 3	Log D (pH 7.4)	−0.80 ± 0.08	−2.84 ± 0.17	−3.13 ± 0.02	−3.35 ± 0.16
Protein binding [%] *n* = 3	30 min	1.6 ± 0.8	1.2 ± 1.1	10.5 ± 1.1	17.7 ± 4.1
	60 min	2.4 ± 1.9	1.3 ± 1.4	23.8 ± 1.4	22.3 ± 2.6
	120 min	1.4 ± 1.1	0.7 ± 0.4	24.1 ± 2.2	20.3 ± 6.1
Serum stability *n* = 2	60 min	>99%	>99%	56%	99%
	120 min	>99%	>99%	39%	98%
	240 min	>99%	>99%	14%	98%

Data are presented as mean ± SD.

**Table 2:** Minimal inhibitory concentrations (MIC) of *A. fumigatus* incubated for 24 h and 48 h with antifungal conjugates at 37 °C **in *Aspergillus* min imal medium (AMM)**. (Values from three biological replicates).

Compound	MIC 24 h		MIC 48 h	
	µg/mL	µM	µg/mL	µM
[Fe]DAFC-NLF-Ac	>16	>12.5	>16	>12.5
[Fe]DAFC-succ-NLF	>16	>12.0	>16	>12.0
HOOC-NLF-Ac	>16	>36.8	>16	>36.8
[Fe]DAFC-L-PAF26	>16	>8.4	>16	>8.4
[Fe]DAFC-D-PAF26	>16	>8.4	>16	>8.4
HOOC-L-PAF26-NH_2_	>16	>16.8	>16	>16.8
H_2_N-D-PAF26-NH_2_	>16	>16.9	>16	>16.9
H_2_N-D-PAF26-Ac	>16	>16.1	>16	>16.1

**Table 3:** MIC of *A. fumigatus* incubated for 24 h and 48 h with antifungal conjugates at 37 °C **in 0.1 × potato dextrose broth (0.1 × PDB**), important numbers are highlighted in bold (Values from three biological replicates).

Compound	MIC 24 h		MIC 48 h	
	µg/mL	µM	µg/mL	µM
[Fe]DAFC-D-PAF26	>16	>8.4	>16	>8.4
H_2_N-D-PAF26-NH_2_	**8**	**8.4**	**8**	**8.4**
H_2_N-D-PAF26-Ac	16	16.1	>16	>16.1

**Table 4:** In vivo stability of different antifungal conjugates radiolabelled with gallium-68. Samples from BALB/c mice 10 min after injection into the tail vein. Values reflect intact conjugate measured by radio-HPLC analysis (expressed as % radioactivity of intact conjugate in relation to metabolites.

	[^68^Ga]Ga-DAFC-NLF-Ac	[^68^Ga]Ga-DAFC-succ-NLF	[^68^Ga]Ga-DAFC-D-PAF26

Blood	86.6%	>99%	>99%
Urine	64.0%	89.4%	57.0%

**Table 5:** Biodistribution of gallium-68 labelled compounds in standard BALB/c mice after 45 and 90 min shown as injected dose per gram tissue (%ID/g). Each value represents three biological replicates, crucial numbers are highlighted in bold. * Data of [^68^Ga]Ga-TAFC are adapted from reference [31].

Organ	[^68^Ga]Ga-DAFC-NLF-Ac		[^68^Ga]Ga-DAFC-succ-NLF		[^68^Ga]Ga-DAFC-D-PAF26		[^68^Ga]Ga-TAFC *	
	45 min	90 min	45 min	90 min	45 min	90 min	30 min	90 min
Blood	0.46 ± 0.10	0.13 ± 0.10	0.41 ± 0.14	0.05 ± 0.03	1.08 ± 0.12	0.40 ± 0.04	1.60 ± 0.37	0.06 ± 0.04
Spleen	0.35 ± 0.10	0.24 ± 0.07	0.23 ± 0.04	0.19 ± 0.03	2.21 ± 0.15	1.97 ± 0.21	0.38 ± 0.11	0.05 ± 0.02
Pancreas	0.21 ± 0.06	0.10 ± 0.06	0.14 ± 0.05	0.06 ± 0.02	0.41 ± 0.02	0.26 ± 0.03	0.42 ± 0.16	0.04 ± 0.01
Stomach	0.69 ± 0.31	2.04 ± 0.74	0.19 ± 0.09	0.11 ± 0.01	0.45 ± 0.17	0.35 ± 0.03	0.77 ± 0.22	0.06 ± 0.02
Intestine	**3.09 ± 0.03**	**4.85 ± 1.78**	**0.40 ± 0.10**	**0.36 ± 0.07**	0.63 ± 0.02	0.56 ± 0.03	1.71 ± 10.04	1.03 ± 0.26
Kidneys	**35.69 ± 1.73**	**39.88 ± 2.66**	**3.27 ± 0.61**	**2.93 ± 0.38**	**149.62 ± 17.99**	**158.18 ± 25.65**	**5.51 ± 1.22**	**1.27 ± 0.39**
Liver	0.85 ± 0.51	0.44 ± 0.05	0.37 ± 0.07	0.30 ± 0.05	**20.79 ± 0.58**	**23.00 ± 0.89**	0.70 ± 0.04	0.15 ± 0.09
Heart	0.22 ± 0.02	0.12 ± 0.11	0.18 ± 0.09	0.05 ± 0.02	0.76 ± 0.02	0.60 ± 0.23	0.67 ± 0.16	0.03 ± 0.01
Lung	0.62 ± 0.01	0.32 ± 0.06	0.61 ± 0.14	0.21 ± 0.05	2.16 ± 0.19	1.31 ± 0.23	1.54 ± 0.27	0.10 ± 0.03
Muscle	0.18 ± 0.08	0.17 ± 0.07	0.08 ± 0.01	0.11 ± 0.14	0.42 ± 0.18	0.28 ± 0.10	0.52 ± 0.25	0.03 ± 0.01
Femur	0.16 ± 0.05	0.59 ± 0.50	1.76 ± 0.17	0.31 ± 0.13	1.47 ± 0.09	1.11 ± 0.16	0.51 ± 0.79	0.15 ± 0.20

Data are presented as mean ± SD.

## Supplementary Materials

The following are available online at https://www.mdpi.com/2309-608X/6/4/367/s1.
