# Supplementary material for: Siderophore Scaffold as Carrier for Antifungal Peptides in Therapy of Aspergillus fumigatus Infections
Source: J Fungi (Basel). 2020 Dec 15;6(4):367. doi: 10.3390/jof6040367 (PMC7765500; doi:10.3390/jof6040367)
Supplement: Supplementary file 1 [file jof-06-00367-s001.pdf]

SUPPLEMENTARY MATERIAL

Siderophore scaffold as carrier for antifungal peptides in therapy of *Aspergillus fumigatus* infections

Joachim Pfister [1], Roland Bata [1], Isabella Hubmann [1], Anton Amadeus Hörmann [1], Fabio Gsaller [2], Hubertus Haas [2] and Clemens Decristoforo [1,*]

MALDI-TOF MS: Matrix-assisted laser desorption/ionization time -of-flight mass spectrometry was performed on a Bruker microflex™ bench-top MALDI-TOF MS (Bruker Daltonics, Bremen, Germany). Samples were prepared on a micorscout target (MSP96 target ground steel BC, Bruker Daltonics) using dried-droplet method and α-cyano-4-hydroxycinnamic acid (HCCA, Sigma-Aldrich, Handels GmbH, Vienna, Austria) as matrix. All spectra were recorded by summarizing 600 laser shots per spot and Flex Analysis 2.4 software was used for data processing.

Analytical RP-HPLC: Reversed-phase (RP) high-perfomance Liquid chromatography (HPLC) analysis was carried out using the following instrumentation: UltiMate 3000 RS UHPLC pump, UltiMate 3000 autosampler, UltiMate 3000 Variable Wavelenght Detector; UV detection at λ = 220nm (Dionex, Germering, Germany) Radio-detector (Gabi Star, Raytest; Straubenhardt, Germany) using Jupiter 5 µM C_{18} 300 Å 150 x 4.6 mm (Phenomenex Ltd. Aschaffenburg, Germany) as column with acetonitrile (ACN)/H_2O/0.1% trifluoroacetic acid (TFA) as mobile phase; flow rate of 1 mL/min;

Gradient A: 0.0–3.0 min 10% ACN, 3.0–16.0 min 10–60 % ACN, 16.0–18.0 min 60% ACN, 18.0–18.1 min 60–10% ACN, 18.1–22.0 min 10% ACN.

Preparative RP-HPLC. Sample purification via RP-HPLC was carried out on a Gilson 322 Pump with a Gilson UV/VIS-155 detector (UV detection at λ = 220 nm) using a PrepFC™ automatic fraction collector (Gilson, Middleton, WI, USA), Eurosil Bioselect Vertex Plus 30 x 8 mm 5 µm C_{18}A 300Å pre-column and Eurosil Bioselect Vertex Plus 300 x 8 mm 5 µm C_{18}A 300Å column (Knauer, Berlin, Germany) and following ACN/H_2O/ 0.1 % TFA gradients with a flow rate of 2 mL/min:

Gradient 1: 0.0–1.0 min 0 % ACN, 1.0–35.0 min 0–50 % ACN, 35.0–36.0 min 50 % ACN, 36.0–36.1 min 50–0 % ACN, 36.1–43.0 min 0 % ACN

Precursor preparation:

[Fe]FsC was produced as previously described [1]. Briefly, supernatant of a *Aspergillus fumigatus* culture mutant strain *ΔsidG* was used according to Schrettl et al [2] to produce [Fe]Fsc and purified using a Reveleris silica flash cartridge (C18; 40µm; 12g; column volume of 18mL; BÜCHI Labortechnik AG, Flawil, Switzerland). [Fe]FsC (30 mg, 38 µmol) was dissolved in 500 µL of water and rocked with 20 µL (0.2 µmol) of acetic anhydride to get [Fe]DAFC after purification with preparative RP-HPLC.
[Fe]DAFC was further used to produce [Fe]DAFC succ by adding an excess of succinic anhydride in DMF, which was also purified by preparative RP-HPLC. [3]

Conjugation of the antifungals:

Synthesis of D-PAF26

The synthesis of the D-PAF26 (H_2N-dArg-dLys-dLys-dTrp-dPhe-dTrp-NH_2) was carried out as previously described.[4] Briefly, 300 mg of Rink Amide resin (189 µmol; 0.63 mmol/g) was used following Fmoc protocols. The amino acid dArg was protected by a Pbf protecting group, whereas for dTrp and dLys Boc protection groups were used. Activation of the carboxylic group was performed by incubation with HATU/HOAt for 10 min at room temperature in DMF using a 3-fold molar excess (567 µmol). After coupling of the last amino acid and removing of the Fmoc group, the peptide was cleavage from the resin with TFA/Triisopropylsilane/H2O (v/v/v; 95/2.5/2.5) for 45 min at room temperature. The crude peptide was precipitated with ice-cold diethyl ether and dissolved in 20% v/v ACN/H_2O. Hereafter the peptide was purified by preparative RP-HPLC to get H_2N-D-PAF26-H_2N. To produce the acetylated form of the peptide, acetic anhydride was added as a last step to the resin coupled peptide. Hereafter the same cleavage and purification procedure was applied to get Ac-D-PAF26-H_2N. To produce L-PAF26, synthesis was performed in the same way, except by using L-amino acids.

Analytical data: H_2N-D-PAF26-NH_2 5.4 mg [5.6 µmol, 96% purity] preparative RP-HPLC gradient 1, t_R = 22.1 min; analytical RP-HPLC gradient A, t_R = 12.2 min; MALDI TOF-MS: m/z [M+H]+ = 949.52 [$C_{49}H_{68}N_{14}O_6$; exact mass: 948.54 (calculated)].

Analytical data: Ac-D-PAF26-NH_2 9.3 mg [9.3 µmol, 96% purity] preparative RP-HPLC gradient 1, t_R = 22.9 min; analytical RP-HPLC gradient A, t_R = 12.4 min; MALDI TOF-MS: m/z [M+H]+ = 991.43 [$C_{51}H_{70}FeN_{14}O_7$; exact mass: 990.55 (calculated)].

Analytical data: H_2N-D-PAF26-COOH 10.8 mg [11.3 µmol, 98% purity] preparative RP-HPLC gradient 1, t_R = 24.0 min; analytical RP-HPLC gradient A, t_R = 12.3 min; MALDI TOF-MS: m/z [M+H]+ = 950.13 [$C_{90}H_{125}FeN_{20}O_{22}$; exact mass: 949.52 (calculated)].

To couple the siderophore to D-PAF26, 7.6 mg (7.8 µmol) of [Fe]DAFC succ was dissolved in DMF and activated with a twofold excess of HATU (6.0 mg, 15.6 µmol) for 10 min at room temperature. Hereafter, the solution was added to the Fmoc free peptide bound to the resin and left to react for 4h and cleavage as described before. Analytical data: [Fe]DAFC-D-PAF26 900 µg [0.4 µmol, 70% purity] preparative RP-HPLC gradient 1, t_R = 31.1 min; analytical RP-HPLC gradient A, t_R = 12.9 min; MALDI TOF-MS: m/z [M+H]+ = 1894.65 [$C_{90}H_{125}FeN_{20}O_{22}$; exact mass: 1893.86 (calculated)].

Analytical data: [Fe]DAFC-L-PAF26 2.5 mg [1.3 µmol, 90% purity] preparative RP-HPLC gradient 1, t_R = 31.1 min; analytical RP-HPLC gradient A, t_R = 13.2 min; MALDI TOF-MS: m/z [M+H]+ = 1896.27 [$C_{90}H_{124}FeN_{19}O_{23}$; exact mass: 1894.84 (calculated)].

Synthesis of NLF

Synthesis of NLF peptide (H_2N-Asn-Leu-D-Phe-COOH) was carried out in the same way as described above. As a starting material 2-Chlortrityl resin was used and the finished peptides were directly coupled to [Fe]DAFC succ ([Fe]DAFC-succ-NLF) or acetylated at the resin and cleavage, to couple the peptide with its free carboxylic group to the amine of [Fe]DAFC ([Fe]DAFC-NLF-Ac).

Analytical data [Fe]DAFC-NLF-Ac 1.2 mg [0.9 µmol, 90% purity] preparative RP-HPLC gradient 1, t_R = 24.0 min analytical RP-HPLC gradient A, t_R = 15.2 min; MALDI TOF-MS: m/z [M+H]+ = 1280.03 [$C_{58}H_{83}FeN_{10}O_{19}$; exact mass: 1279.51 (calculated)].

Analytical data [Fe]DAFC-succ-NLF 1.5 mg [1.1 µmol, 88% purity] preparative RP-HPLC gradient 1, t_R = 22.0 min analytical RP-HPLC gradient A, t_R = 14.7 min; MALDI TOF-MS: m/z [M+H]+ = 1338.34 [$C_{60}H_{85}FeN_{10}O_{21}$; exact mass: 1337.52 (calculated)].

Iron free conjugates:

For iron removal half of the conjugate was stirred in 1 mL of 100 mM Na_2EDTA solution for 2 hours to get the iron free compound, which was subsequently purified by RP-HPLC and dried with vacuum to get a white solid powder.

DAFC-D-PAF26: Analytical data DAFC-D-PAF26 0.3 mg [0.2 µmol, 90% purity] analytical RP-HPLC gradient A/B, t_R = 13.2 min; MALDI TOF-MS: m/z [M+H]+ = 1841.65 [$C_{90}H_{128}N_{20}O_{22}$; exact mass: 1840.95 (calculated)].

DAFC-L-PAF26: Analytical data DAFC-L-PAF26 740 µg [0.2 µmol, 95% purity] analytical RP-HPLC gradient A, t_R = 12.8 min; MALDI TOF-MS: m/z [M+H]+ = 1841.65 [$C_{90}H_{128}N_{20}O_{22}$; exact mass: 1840.95 (calculated)]

DAFC-NLF-Ac: Analytical data DAFC-NLF-Ac 0.3 mg [0.2 µmol, 80% purity] analytical RP-HPLC gradient A, t_R = 14.8 min; MALDI TOF-MS: m/z [M+Na]+ = 1250.02 [$C_{58}H_{86}N_{10}O_{19}$; exact mass: 1226.69 (calculated)].

DAFC-succ-NLF: Analytical data DAFC-succ-NLF 0.5 mg [0.2 µmol, 80% purity] analytical RP-HPLC gradient A, t_R = 12.5 min; MALDI TOF-MS: m/z [M+Na]+ = 1309.06 [$C_{60}H_{88}N_{10}O_{21}$; exact mass: 1284.61 (calculated)].

1. Pfister, J.; Summer, D.; Petrik, M.; Khoylou, M.; Lichius, A.; Kaeopookum, P.; Kochinke, L.; Orasch, T.; Haas, H.; Decristoforo, C.; et al. Hybrid Imaging of Aspergillus fumigatus Pulmonary Infection with Fluorescent, 68Ga-Labelled Siderophores. *Biomolecules* **2020**, *10*, 168.
2. Schrettl, M.; Bignell, E.; Kragl, C.; Sabiha, Y.; Loss, O.; Eisendle, M.; Wallner, A.; Arst, H.N.; Haynes, K.; Haas, H. Distinct roles for intra- and extracellular siderophores during Aspergillus fumigatus infection. *PLoS Pathog.* **2007**, *3*, 1195–207.
3. Pfister, J.; Lichius, A.; Summer, D.; Haas, H.; Kanagasundaram, T.; Kopka, K.; Decristoforo, C. Live-cell imaging with Aspergillus fumigatus-specific fluorescent siderophore conjugates. *Sci. Rep.* **2020**, *10*, 15519.
4. Pfister, J.; Summer, D.; Rangger, C.; Petrik, M.; von Guggenberg, E.; Minazzi, P.; Giovenzana, G.B.; Aloj, L.; Decristoforo, C. Influence of a novel, versatile bifunctional chelator on theranostic properties of a minigastrin analogue. *EJNMMI Res.* **2015**, *5*, 74.
